# 

**Published:** 1883-11

**Authors:** 


ORIGINAL COMMUNICATIONS:

Difficult Dentition and the use of the Gum Lancet. J. Morgan Howe. 573
Something about Engines and Hand-Pieces. Norman W. Kingsley.... 582
Anteseptic Treatment of Diseases of the Dental Pulp. Dr. Rosenthal. 585
The Causes of the Failure of Gold as a Filling Material. A. A. Blount 588
Notes on Operative Dentistry and its Advancement. Geo. H. Perine... 591

REPORTS OF SOCIETY MEETINGS:

Report of the Meeting of the Pennsylvania State Dental Society....... 594
Report of the Meeting of the American Dental Society of Europe.... 602
First Annual Meeting of the New England Dental Society ........... 612

CORRESPONDENCE. ........................................ 615

EDITORIAL:

The Prone Position for the Administration of Anæsthetics........... 618
The Lips of Patients........................................ 619
Laboratory Lathes........................................ 620
Caries of Human Teeth........................................ 621
Machinery........................................ 621
Our Journal........................................ 621

NEW APPLIANCES AND MATERIALS:

Abbott's Scissors........................................ 622
Oxy-Phosphate Cement........................................ 623
Pearson's Appointment Book........................................ 623
Kearsing's Foil........................................ 623

CURRENT NEWS AND OPINION:

Difficult Dentition and the use of the Gum Lancet.................. 624
A Clear Diagnosis........................................ 624
Wood Wool........................................ 625
Hot Water in the Treatment of Inflammation of the Mucous Membranes 625
Ignorant Teachers........................................ 626
Death following Extraction of a Tooth........................................ 626
Dental Convention........................................ 626
Brooklyn Dental Society........................................ 627
A Just Criticism........................................ 627
Insanity in the United States........................................ 627

